# Are schoolchildren less infected if they have good knowledge about parasitic worms? A case study from rural Côte d’Ivoire

**DOI:** 10.1186/s12889-018-5776-z

**Published:** 2018-08-02

**Authors:** Marta S. Palmeirim, Mamadou Ouattara, Clémence Essé, Véronique A. Koffi, Rufin K. Assaré, Eveline Hürlimann, Jean T. Coulibaly, Nana R. Diakité, Kouassi Dongo, Bassirou Bonfoh, Jürg Utzinger, Eliézer K. N’Goran, Giovanna Raso

**Affiliations:** 1 Swiss Tropical and Public Health Institute, Basel, Switzerland; 2 University of Basel, Basel, Switzerland; 3 Institute of Hygiene and Tropical Medicine, New University of Lisbon, Lisbon, Portugal; 4 Unité de Formation et de Recherche Biosciences, Université Félix Houphouët-Boigny, Abidjan, Côte d’Ivoire; 5 Unité de Formation et de Recherche Sciences de l’Homme et de la Société, Université Félix Houphouët-Boigny, Abidjan, Côte d’Ivoire; 6 Centre Suisse de Recherches Scientifiques en Côte d’Ivoire, Abidjan, Côte d’Ivoire

**Keywords:** Soil-transmitted helminths, *Schistosoma mansoni*, Health education, Knowledge, Risk perception, Awareness, Côte d’Ivoire

## Abstract

**Background:**

Parasitic worms (helminths) are common infections in low- and middle-income countries. For most helminth species, school-aged children are at highest risk of infection and morbidity, such as impaired cognitive and physical development. Preventive chemotherapy is the current mainstay for helminthiases control. Sanitation improvement and hygiene-related education are important complementary strategies, which act by altering children’s behaviour. However, little is known about the effect of improved knowledge on the risk of helminth infection. The aim of this study was to assess the potential influence of knowledge that children acquired at home or in school, without any specific health education intervention, on helminth infections.

**Methods:**

In May 2014, we conducted a cross-sectional survey in western Côte d’Ivoire. A total of 2498 children, aged 9-12 years, were subjected to three consecutive stool examinations using duplicate Kato-Katz thick smears to determine infections with soil-transmitted helminths and *Schistosoma mansoni*. Additionally, children were interviewed to assess their knowledge about helminth infections. Four knowledge scores were constructed by factor analysis; one, reflecting general knowledge about helminths and three manifesting helminth species-specific knowledge. The effect of general and specific knowledge on children’s helminth infection status was determined using meta-analysis.

**Results:**

Children who scored high in the hookworm-specific knowledge were less likely to be infected with hookworm but no association was found for the other helminth species. Moreover, greater general knowledge was not associated with lower odds of being infected with any helminth species. Most of the children interviewed believed that the effect of preventive chemotherapy is permanent, and hence, re-treatment is not necessary.

**Conclusions:**

Specific knowledge about different types of helminths might not suffice to induce behavioural change which in turn reduces infection and reinfection with helminths. Health education interventions should strive to strengthen the perception of risk and to clarify the true benefit of preventive chemotherapy.

## Background

Parasitic worms, particularly soil-transmitted helminths and schistosomes, are widespread. Indeed, an estimated 1.5 billion people are infected, mainly in low- and middle-income countries [1, 2]. Helminthiases cause considerable burden, including physical and intellectual growth retardation among preschool- and school-aged children [3, 4]. Despite their negative impact on public health, educational attainment, social and economic development, helminthiases are often neglected [5, 6]. Helminthiases account for the largest burden among the neglected tropical diseases [7] and are governed by inadequate water supply and sanitation, improper hygiene habits, crowded living conditions, difficult access to health care and low levels of education [2, 8].

Key strategies recommended by the World Health Organization (WHO) for the prevention, control and elimination of helminthiases include preventive chemotherapy (periodic deworming of school-aged children and other high risk groups), and water and sanitation supply supported by personal hygiene and health education [9]. Although effective in the short-term, preventive chemotherapy does not prevent reinfection and, providing adequate sanitation and clean water alone, do not necessarily mean these measures will be adequately used, in which case prevalence and intensity of infection may not decrease in the long run [10–12]. Improved knowledge through health and hygiene-related education might induce behavioural change, which in turn might result in reduced exposure to helminth infections, thus avoiding or delaying reinfection [13–15]. Indeed, the gains of preventive chemotherapy, water supply and sanitation improvement can be reinforced through enhanced knowledge [16, 17].

Although this topic has gained traction in recent years, relatively little research has investigated the effect of health education interventions on the prevalence, intensity and re-infection patterns of soil-transmitted helminths [18–21]. However, results reported in the literature are inconsistent. While some studies have found that health education was associated with a decrease in helminth infection [17, 18, 21], other studies failed to detect such an association [19, 20]. This discrepancy might be explained by specific geographic, social, cultural and infrastructure conditions under which the effectiveness of health education was evaluated. Hence, there is a need for empirical studies assessing the effect of knowledge on helminth infection rates. Research pursued in different settings will deepen our understanding of how improved knowledge results in reduced rates of helminth infection.

A limitation of the available information on the effect of knowledge on helminth infection is that most studies carried out thus far focused on the follow-up of specific health education interventions, which are usually meant to not only increase knowledge but also to raise awareness and demystify local myths [17, 18, 20]. However, typically, health-related knowledge that children receive does not come from such specific interventions, but is rather “background” knowledge obtained at home or in school. The level of this “background” knowledge is likely to vary from one child to another and across settings.

In the current study, we assessed the importance of “background” knowledge that children acquired at home or in school, without any specific health education intervention on helminth infections. Our hypothesis was that children’s “background” knowledge regarding soil-transmitted helminths and *Schistosoma mansoni* infections influences their odds of infection, and hence, children with adequate knowledge have better health and hygiene-related behaviours, which in turn results in lower helminth infection levels.

## Methods

### Study site and study design

**Fig. 1:**
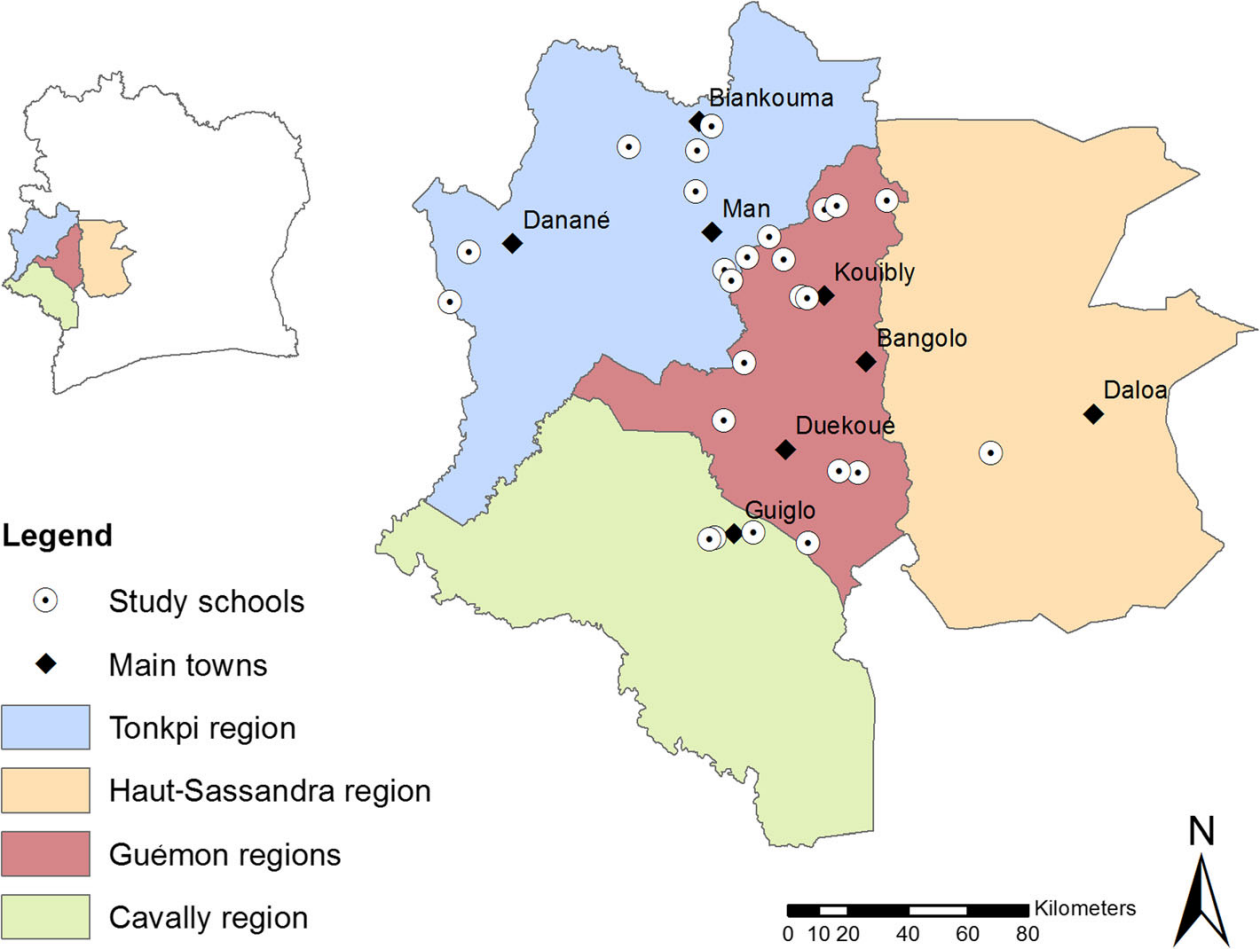
Map of Côte d’Ivoire with the four regions and the 25 schools included in the study

The study was carried out in four regions of western Côte d’Ivoire: Cavally, Guémon, Haut-Sassandra and Tonkpi (Fig. 1). In this part of Côte d’Ivoire, people are mainly engaged in subsistence agriculture [22]. Previous studies in this area have shown that *S. mansoni* and hookworm infections are highly endemic [23, 24]. The study was implemented in 25 schools that are part of a large 5-year operational research project pertaining to sustaining the control of schistosomiasis, facilitated by the Schistosomiasis Consortium for Operational Research and Evaluation (SCORE) [25, 26]. Children in these schools have been receiving an annual dose of praziquantel (40 mg/kg) and albendazole (400 mg) since 2012. An observation of sanitary conditions performed one year after the implementation of the current study showed that, among the 25 schools investigated, soap for washing hands after defecation was only available in three schools (12%), access to clean water from a pump was available in 10 schools (40%) and latrines were available in 17 schools (68%).

In May 2014, about 100 children, aged 9–12 years, were selected in each of the 25 schools. The study consisted of two surveys: (i) a cross-sectional parasitological survey to assess children’s infection with helminths; and (ii) a questionnaire survey to determine children’s knowledge about helminthiases. After the parasitological survey, all school-aged children (5–15 years) of the study localities, including those not participating in our study, were treated with albendazole (400 mg) against soil-transmitted helminthiasis and praziquantel (40 mg/kg) against schistosomiasis by the national control programme.

### Parasitological survey

Each participant was provided with three empty plastic containers that contained unique identification numbers and was invited to submit three stool samples over consecutive days. Stool samples were transferred to nearby laboratories where duplicate Kato-Katz thick smears were prepared on microscope slides from each stool sample. The first set of duplicate slides was examined on the same day under a light microscope, whereas the second set of slides was only examined several days later. The main reason why the second set of slides was examined later was the sheer amount of Kato-Katz thick smears to be examined, coupled with the fact that the study was embedded in a larger SCORE project with particular emphasis on *S. mansoni* [25]. Slides were examined by experienced laboratory technicians using a standard operating procedure [27, 28]. All helminth eggs were enumerated and recorded for each species separately. Results from both slides were pooled for each parasite species. Quality control was done on 10% of the Kato-Katz thick smears.

### Questionnaire survey

Children’s knowledge about health-related hygiene, placing particular emphasis on *S. mansoni* and soil-transmitted helminth infections, their treatment, prevention, symptoms and modes of transmission, were assessed. We also explored children’s attitudes and practices regarding hygiene, and probed for cultural beliefs linked to helminth infections. To allow a combination of qualitative and quantitative data, the questionnaire was designed in a semi-structured manner, including a mixture of open- and closed-ended questions. The questionnaire was administered in French, which is the official language of Côte d’Ivoire and is taught in schools. However, there were a few children who did not speak French. For these non-French speaking children, questions were translated by a local interpreter who was part of the survey team. The questionnaire was pre-tested in a school not otherwise involved in the survey to critically determine its suitability regarding duration, content and comprehension. Interviewers were trained and received instructions concerning the questionnaire and its administration. All children were interviewed individually. The administration of the questionnaire usually lasted between 15 and 30 min.

### Statistical analysis

Questionnaire data were double entered and cross-checked in EpiInfo version 3.5.3 (Centers for Disease Control and Prevention; Atlanta, GA, USA). On the spot, parasitological data were entered into smartphones and transferred onto a central server (Open Data Kit) hosted by the SCORE secretariat at the Task Force for Global Health (Decatur, GA, USA) [25, 26]. The questionnaire and parasitological data were combined into a single master file for subsequent statistical analysis. We used STATA version 10.1 (StataCorp. 2007; College Station, TX, USA) and Quantitative Parasitology version 3.0 [29].

The number of helminth eggs per gram of stool (EPG) was employed as proxy for infection intensity [30]. Infection intensity was grouped into light, moderate and heavy, according to WHO guidelines [30].

Factor analysis was used to create two types of scores: (i) a general knowledge score, which expresses children’s knowledge regarding helminth infections in general; and (ii) helminth species-specific scores, which estimates the participant’s knowledge regarding a specific helminth species. To test whether general knowledge or helminth species-specific scores influenced the risk of infection, a meta-analysis (risk ratio (RR)-based) was performed for each helminth separately as well as for any helminth [31]. Of note, *Ascaris lumbricoides* and *Trichuris trichiura* were pooled because of the low prevalence and similar transmission pathways. To assess whether knowledge influenced the intensity of infection, the helminth species-specific median EPG were compared between more and less knowledgeable groups of children, using Kruskal-Wallis tests.

Because the data are clustered (by school), to test whether sex or age group (9–10 years or 11–12 years) influenced how knowledgeable children were, we used generalized estimated equations (GEE). The exchangeable correlation matrix was chosen because the order of sampling of children does not affect the correlation. Since the data are binary (i.e. more knowledgeable and less knowledgeable), a logistic link function was applied. The same method was used to test whether the infection rates were influenced by sex or age group.

Since not all participants replied to the questionnaire and not all provided three stool specimens, two different cohorts will be mentioned throughout the results section. The first cohort consists of all children who responded to the questionnaire but provided less than three stool specimens, and hence, were used when presenting questionnaire results alone. The second cohort consists of those children who had complete data records (i.e. responded to the questionnaire and provided three stool samples), used when testing association between knowledge and infection.

#### Knowledge scores

Questionnaire surveys might include questions that are redundant or inadequate to quantify knowledge. Factor analysis is a useful technique to explore which of the questions best measure knowledge [32]. This method allows the identification of the questions that are particularly prone to reveal knowledge, as well as those that should either be discarded because they are uninformative, or pooled to minimize redundancy [33]. Factor analysis loadings represent the level of association between a variable and each factor (for the purpose of our analysis this factor is knowledge) and are used in the final interpretation of the analysis. Higher loadings represent a stronger association between a question and a child’s knowledge.

We discarded questions which had loadings lower than 0.4 on the factor representing knowledge. The remaining questions were used to create a summated scale, which only included variables that loaded highly on the factor and excluded those that had little effect. Hence, this scale consisted on the combination of several questions into a single measure of knowledge. We assessed the internal consistency of the summated scale, i.e. whether the questions in the scale had a strong relationship between each other. We used the Cronbach alpha (α), which is a measure of scale reliability. The relationship with the latent variable (knowledge) was considered strong if α ≥0.6 [34]. The results of the factor analysis suggested that several questions should be combined (Table 1). For example, in the case of the symptom question “what are the symptoms of intestinal worms?”, the three sub-questions “do they cause diarrhoea?”, “do they cause a belly ache?” and “do they cause fatigue?” were combined into a single score. Other questions were combined using the same approach. Overall, six main questions were selected for the construction of this score. In all cases, the participant was considered to have an overall positive answer when he/she replied correctly to over 65% of the sub-questions. This cut-off was defined as a percentage because each of the six selected questions had a different number of sub-questions making it impossible to define a single number of sub-questions that should be answered correctly in order to be considered knowledgeable regarding that question. The general knowledge score of each participant was calculated by summing the points gained in all six main questions selected by the aforementioned methodology (Table 1). A child was considered to have high general knowledge when he/she replied correctly to at least five of the six questions. This way, participants were separated into two groups based on the results of the questionnaire: good knowledge (five or six correct answers) and less knowledgeable (four or less correct answers).

**Table 1:** Explanation of the general knowledge scoring process

Question	Possible answers	Points
Are these correct modes of helminth transmission?	• Eating without washing hands	+ 1 point if ≥ 5 questions were answered affirmatively
	• Drinking from the backwater	
	• Playing in dirty water	
	• Walking on garbage	
	• Not wearing shoes	
	• Playing in the soil	
	• Eating raw vegetables/fruits	
Are these incorrect modes of transmission (myths)?	• Eating too much sugar	- 1 point if ≥ 2 questions were answered affirmatively
	• Eating too much fruit	
	• Eating rotten food	
Are these symptoms of intestinal worm infections?	• Fatigue	+ 1 point if ≥ 5 questions were answered affirmatively
	• Hard time concentrating	
	• Hard time thinking	
	• Diarrhoea	
	• Not growing well	
	• Abdominal pain	
	• Lack of appetite	
Should you defecate in latrines?		+ 1 point if answered affirmatively
Should you open air defecate?		- 1 point if answered affirmatively
Should you drink from the backwater?		- 1 point if answered affirmatively
		Total general knowledge score

The sum of all points was considered for stratification of children into good knowledge (≥ 5 points) or less knowledgeable (< 5 points)

The questionnaire included a few questions which reflected the interviewee’s knowledge regarding the transmission pathway of a specific helminth species. Using these questions, we created three parasite-specific scores: (i) *A. lumbricoides* and *T. trichiura* combined; (ii) hookworm; and (iii) *S. mansoni*. For the *A. lumbricoides* and *T. trichiura* combined score, three questions were selected, and hence, a child was considered to have good knowledge if he/she answered correctly to two out of the three questions. With regard to the *S. mansoni* and hookworm scores, a child was considered to have good knowledge if he/she answered correctly to one specific question related to each parasite.

## Results

### Operational results

**Fig. 2:**
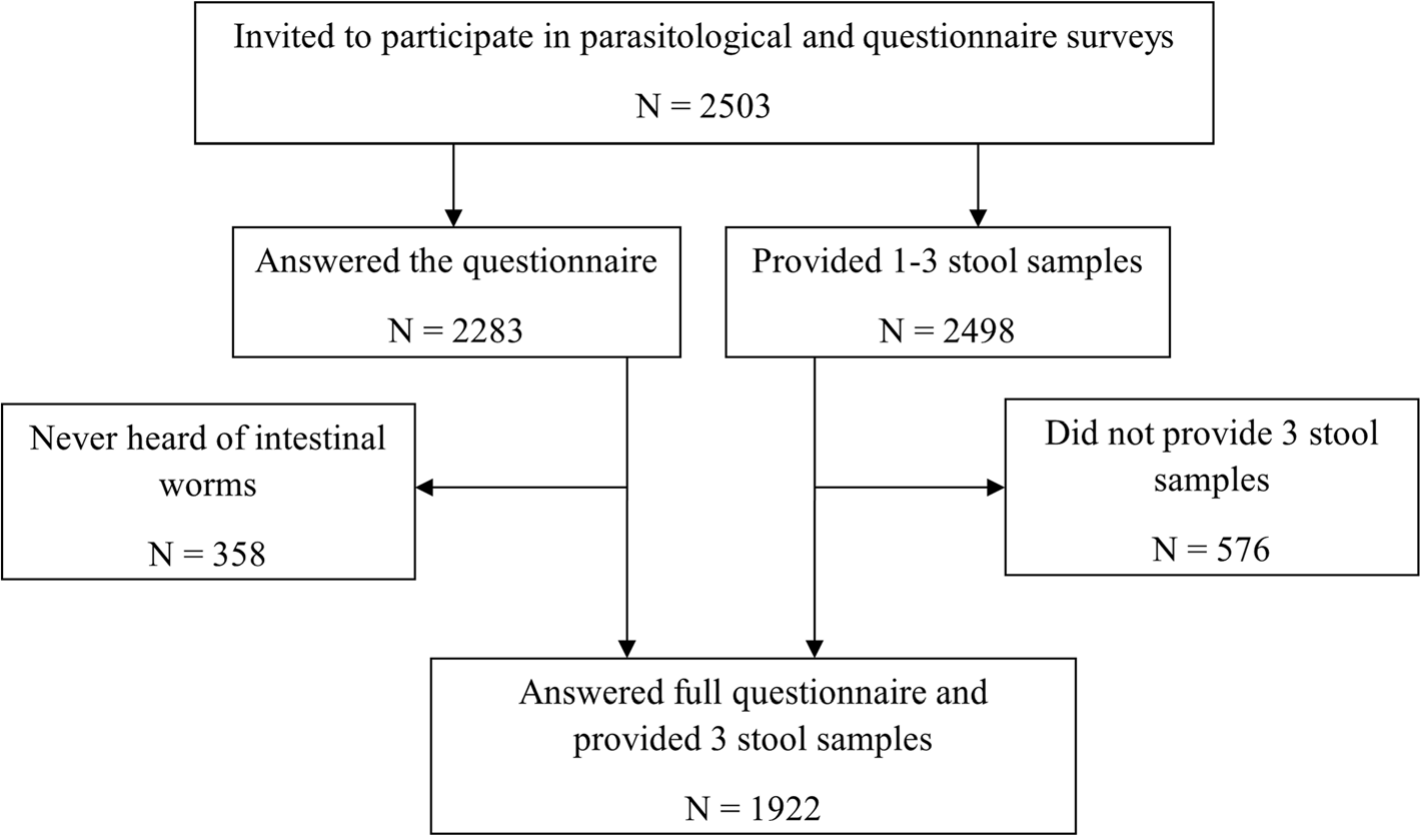
Participation flow chart. Children who never heard of parasitic worms did not reply to the whole questionnaire and therefore were not included in the analysis related to knowledge

Stool samples were obtained from 2498 children, of whom 1922 (76.9%) provided three, 472 (18.9%) two and 104 (4.2%) one stool sample. Questionnaire results were available from 2283 children. However, 358 children (15.7%) had never heard of parasitic worms, and hence, they were only included in the parasitological analysis (Fig. 2). Of the 1922 children with complete parasitological data, 852 (44.3%) were females. In terms of age, 1035 children (53.8%) were aged 9–10 years, while the remaining 887 children (46.2%) were 11 or 12 years old. The prevalence of hookworm, *S. mansoni*, *T. trichiura* and *A. lumbricoides* was 14.4, 8.9, 2.6 and 1.6%, respectively.

### Questionnaire survey

Based on the calculated general knowledge scores, only a third (34.8%) of the children was considered to have a good knowledge (Table 2).

**Table 2:** Percentage of children answering correctly to the general knowledge questions during the cross-sectional survey in 25 schools of western Côte d’Ivoire in May 2014 (*N* = 2283)

Number of correct answers	% of children	Knowledge category
0	0.3	Less knowledgeable
1	2.8	
2	9.1	
3	13.2	
4	39.8	
5	30.6	Good knowledge
6	4.2	

We found that sex was not associated with knowledge about helminth infections (GEE, *p* > 0.05 for any helminth). However, older children were significantly more knowledgeable for each of the four helminth species (GEE, *p* < 0.001 for each helminth species). Table 3 shows the percentage of children who answered correctly each of the questions used to create the helminth species-specific scores.

**Table 3:** Percentage of children answering correctly to each of the questions used for the parasite-specific scores during a cross-sectional survey in 25 schools of western Côte d’Ivoire in May 2014 (*N* = 2283)

Helminth infection	Question	% of children answering correctly
*S. mansoni*	Can you become infected if you play in dirty water?	61.8
Hookworm	Should you defecate in latrines?	82.5
*A. lumbricoides* and *T. trichiura* combined	Can you become infected if you do not wash your hands before eating?	65.2
	Can you become infected if you drink water from the river?	45.5
	Can you become infected if you eat unwashed fruits or vegetables?	55.4

Although not included in the score, the questionnaire revealed that 85% of children believed that there is efficient treatment available and of those, 86% believed that it prevents from re-infection.

### Influence of knowledge on helminth infections

**Fig. 3:**
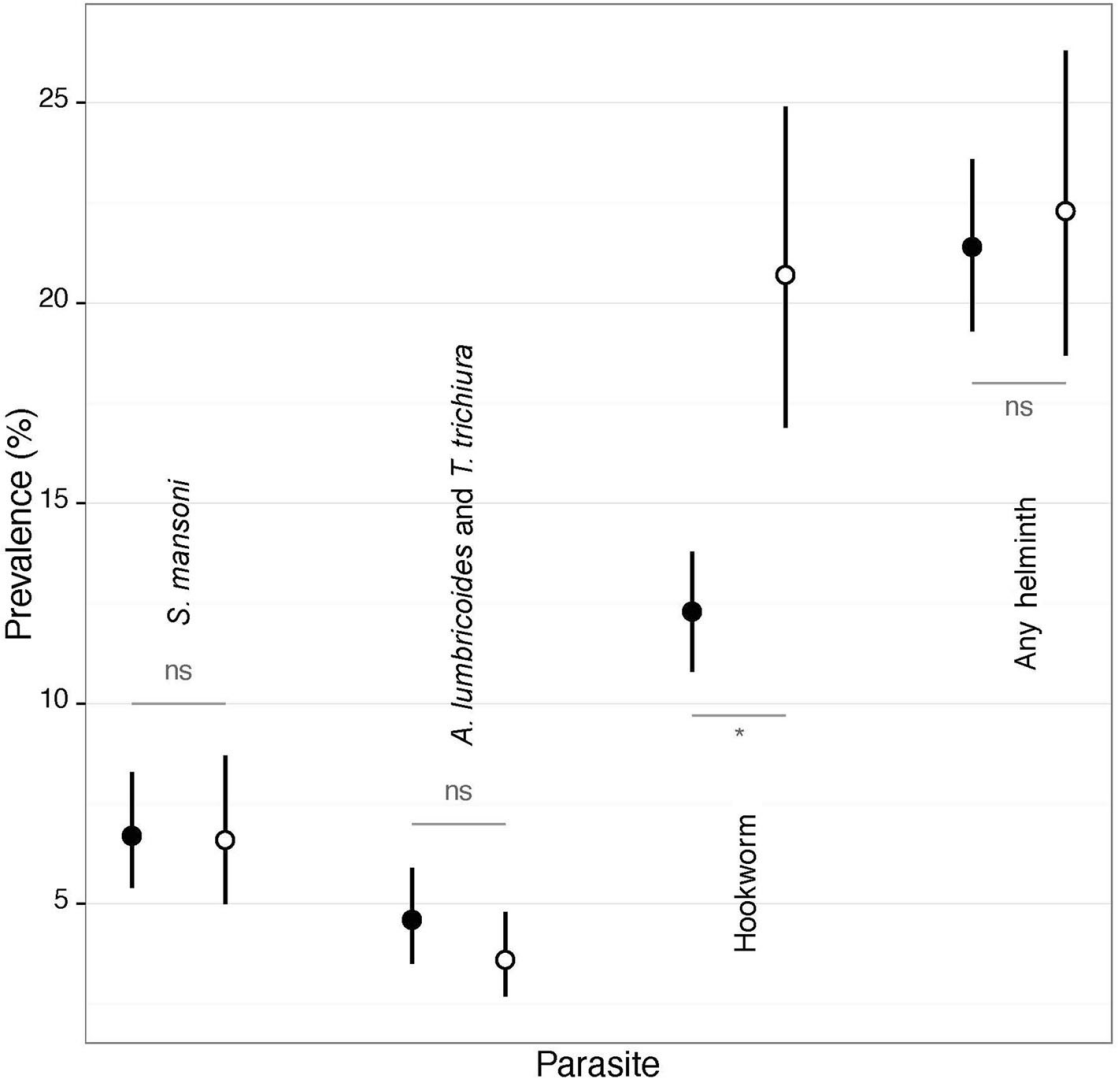
Prevalence of helminth infection in children with more (black dot) and less (white dot) knowledge of the modes of transmission of each helminth species. ns = non-significant, * = *P* < 0.05

**Fig. 4:**
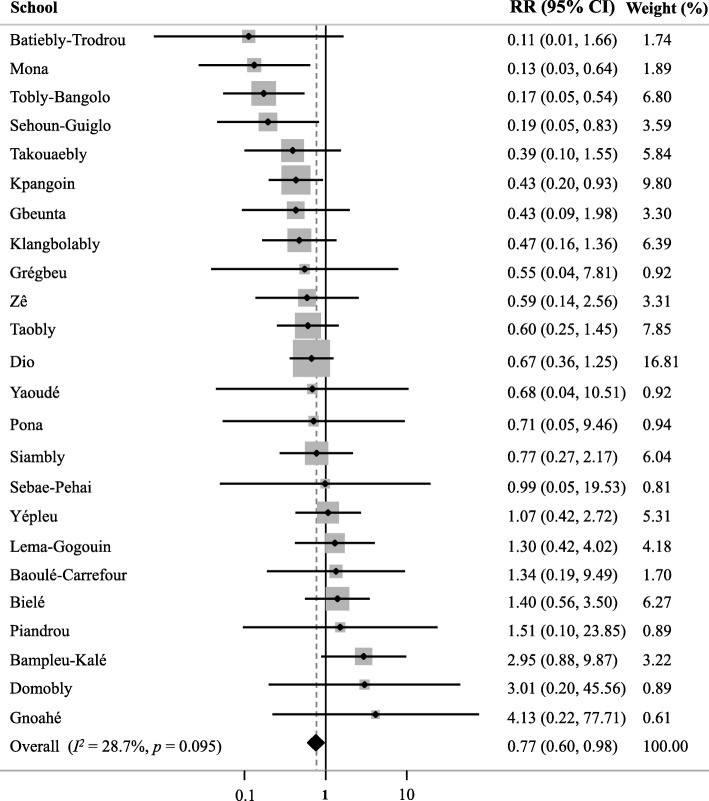
Forest plot representing the overall and school-level effects of knowledge about hookworm on the infection RR

Figure 3 depicts the effect of knowledge about modes of helminth transmission on the odds of infection. We found no significant influence of general knowledge on the infection rate by any of the four helminth species (meta-analysis, *p* = 0.839). We also observed that answering correctly to questions associated with *S. mansoni* did not influence its prevalence (meta-analysis, *p* = 0.884); hence, children who knew that playing in dirty water is a risk factor for infection showed similar infection rates than those who were less knowledgeable. The same applied to the questions related to *A. lumbricoides* and *T. trichiura* (meta-analysis, *p* = 0.265); children who had a good knowledge about these two helminth species were not found to be less infected. However, in the case of hookworm, specific knowledge that using a latrine for defecation is an appropriate behaviour was associated with a significantly lower odds of hookworm infection (meta-analysis, *p* = 0.034) (Fig. 3). The forest plot shows that, despite some variability among schools, there is a tendency that a low RR is associated with a higher knowledge level (Fig. 4).

In terms of infection intensity, we found that knowledge about a specific helminth species was not associated with an overall lower level of infection; hence, children who answered correctly to questions related to specific helminth species were not infected with a lower intensity than those who did not answer correctly (Kruskal-Wallis, *p* > 0.05 for any helminth). The same observation was made for general knowledge; higher general knowledge scores were not associated with lighter infection intensities with any helminth (Kruskal-Wallis, *p* > 0.05).

## Discussion

Children with good hygiene knowledge (e.g. use of latrine for defecation) had lower hookworm prevalence than their counterparts who lacked such knowledge. It is conceivable that children who perceive open defecation as a negative behaviour are more likely to use latrines. Such children might also more often wear shoes, and hence, are less prone to stepping on contaminated soil while visiting areas that are convenient to defecate near villages. Our results are encouraging and in line with experiences from a health education intervention gained in the People’s Republic of China [17]. Indeed, the implementation of an animated cartoon had a positive effect on children’s behaviour; it reduced soil-transmitted helminth infections by 50% demonstrating that, at least in some contexts, better knowledge can effectively improve children’s behaviour. However, in the current study, although we found a significant relationship between hygiene knowledge and hookworm infection, children who scored higher in general knowledge had similar infection prevalence of *S. mansoni* and *A. lumbricoides*-*T. trichiura* combined as their less knowledgeable counterparts. Our findings suggest that knowledge is not necessarily effective in inducing changes in behaviour and health-related practices that prevent helminth infection. Hence, even when children knew how to avoid helminth infections, they did not necessarily change their hygiene- and health-related practices. Other groups have also described situations in which acquiring knowledge did not readily translate into behavioural changes [35–38].

How can these observations be explained? First, children may underestimate the risk associated with helminth infections because they are not fully aware of their potential severity or of their long term consequences. In fact, most of the children interviewed (85%) believed that efficacious treatment is available and that, once treated, they cannot become re-infected (86%). Such a misconception, which may result from confusion with the long-term protection by vaccination, might lower the perceived risk associated with helminth infections, and hence, reducing the incentive to avoid risky behaviours. Overcoming such misconceptions is, therefore, critical to initiate a change in behaviour. The way knowledge is being transmitted to children does not seem to properly convey the potential danger of these infections, and hence, does not instigate great urge to correctly apply it. The benefits and shortcomings of preventive chemotherapy should be explained more thoroughly in school health programmes. It may be of value to encourage teachers to stimulate children, on a frequent basis, to apply their knowledge as well as remind them of the consequences of not doing so.

Second, the fact that children do not necessarily change their hygiene- and health-related behaviour in response to a deeper understanding of the epidemiology of helminthiases might be explained by a lack of access to clean water and improved sanitation, which obviously represents a barrier between knowledge and practice. For instance, even if children are aware of the risks of getting into contact with contaminated water sites, they will continue to do so until there are alternatives, as shown by Schall [36]. Children are unlikely to give up recreational bathing and playing in a river or a lake on a hot day just because someone told them they may become sick. A study by Kosinski and colleagues in Ghana included the construction of a safe water recreation area [39]. One year after the installation of this water recreation area, the prevalence of *S. haematobium* in children had decreased significantly, illustrating the importance of providing a safe alternative for recreational water activities, in addition to improved knowledge. Increasing the population’s knowledge and wish to improve hygiene and health-related behaviours without providing the conditions to put the new knowledge into practice may disempower and frustrate people [38]. Hence, efforts are warranted to ensure that the recommended behaviours can be practiced. In schools, it is important to find strategies that make teachers feel responsible and motivate them to ensure there is always easily available water and soap in their schools and that latrines are clean and being used correctly. Still, it is important to note that the simple provision of sanitation facilities is not always sufficient. Studies have illustrated situations where available facilities were not widely used, and hence, were not associated with lower helminth infection rates [11]. Whenever facilities are available, helminth infections will depend on consistent use and good maintenance of the facilities [14]. Indeed, in Zambia, Thys and colleagues found that bad smell, presence of flies, inadequate maintenance and challenges of waste disposal explained a continuing preference for open defecation [40].

Third, unlike prior research in the People’s Republic of China [17], we did not find a consistent link between knowledge and helminth infection rates. In our study in Côte d’Ivoire, deeper knowledge did not automatically translate into a decrease of helminth infections. Social and cultural differences may play a role in how well behaviour change is achieved and should, thus, be taken into account in the design of health education interventions. Social pressure may be a strategy to motivate children to start following the health and hygiene-related recommendations more often. Gyorkos and colleagues suggested that social marketing at the community level as well as further involvement of teachers and parents could potentially maximize changes in behaviour leading to a reduction of infection rates [20].

### Strengths and limitations of the study

Unlike previous studies, which aimed at determining the effect of health education interventions on knowledge and helminth infection, the present study focused on the influence of background knowledge on helminth infection. This unique perspective allowed us to identify the type of knowledge, which is most associated with soil-transmitted helminths and *S. mansoni* and, thus, reinforce setting-specific messages for health education interventions. Our study also contributed to a deeper understanding of strategies that are insufficiently effective, and hence, must be reinforced by other strategies. An additional strength of our study resides in the creation of a general knowledge score. In order to identify the most suitable questions for assessing knowledge, we pursued a factor analysis. This method identifies those questions that should not be included and turns the score into a more reliable one.

A limitation of our study is related to the low prevalence of some helminth species, most importantly *A. lumbricoides* and *T. trichiura*. We employed repeated stool sampling and prepared two Kato-Katz thick smears from each stool specimen to increase diagnostic sensitivity, and yet, the prevalence of both *A. lumbricoides* and *T. trichiura* was below 3%. Of note, from the duplicate Kato-Katz thick smears produced, only one was microscopically examined the same day, whilst the second slide was examined only after several days. Hence, the true prevalence of hookworm might have been underestimated, not only because all infections were of light intensity and therefore remained more likely undetected compared to moderate and heavy infections [41], but also because hookworm eggs tend to disintegrate rapidly after preparation of thick smears. Nonetheless, we found a significant association between knowledge and infection rate with hookworm.

## Conclusions

Taken together, children’s knowledge of *S. mansoni*, *T. trichiura* and *A. lumbricoides* varied, yet the infection rates of the most knowledgeable children were similar to less knowledgeable children. However, we showed that children who were aware of the risk of open defecation were less likely to be infected with hookworm. This result is encouraging, as it suggests that knowledge has the potential to translate into behavioural changes that reduce the risk of infection. Being the main source of most health education messages that children receive, we find that schools should (1) provide an opportunity to apply this health and hygiene-related knowledge and (2) increase children’s awareness and willingness to improve their hygiene behaviours. If schools fail to fulfil one or both of these two key functions, which is often the case, they will inevitably diminish the school’s potential to improve children’s health. Education initiatives to complement the role of schools may gain from prior evaluation of the level of knowledge of children. In situations like that of our study, where children already had a fair background knowledge, it may be advantageous to concentrate most of those efforts on implementing strategies to convert knowledge into behavioural changes. As a next logical step we suggest concrete activities to improve preventive behaviour for the other parasites in order to decrease their infection rates.

## Abbreviations

EPG: Eggs per gram of stool
GEE: Generalized estimating equations
RR: Risk ratio
SCORE: Schistosomiasis Consortium for Operational Research and Evaluation
WHO: World Health Organization
